# Temperature and lipid composition differentially regulate KRAS assemblies on membranes

**DOI:** 10.1039/d6cc01853j

**Published:** 2026-05-05

**Authors:** Ji Kang, Elena Scott, Sangho D. Yun, Virginia K. James, Jing-Yuan Chang, Hanieh Bahramimoghaddam, James Downing, Kacie Evans, David H. Russell, Arthur Laganowsky

**Affiliations:** a Department of Chemistry, Texas A&M University College Station TX 77843 USA ALaganowsky@chem.tamu.edu

## Abstract

RAS GTPases oligomerize on membranes to regulate signaling, but factors governing this process remain unclear. Using variable-temperature native mass spectrometry and NanoBiT assays, we show KRAS dimerization is lipid- and temperature-dependent, increasing at higher temperatures, whereas NRAS is unaffected. These results indicate entropy-driven KRAS assembly and reveal isoform-specific mechanisms of membrane organization.

## Abstract 2

Variable-temperature native MS reveals lipid-selective, temperature-dependent RAS dimerization on membranes, uncovering isoform-specific assembly behavior that may guide therapeutic strategies targeting RAS oligomers.

## Graphical abstract

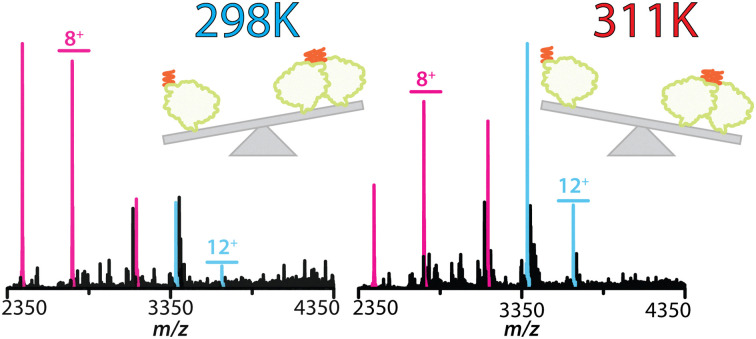

The RAS (rat sarcoma viral oncogene) proteins are among the most frequently mutated oncogenes in cancer, with activating mutations present in nearly one-third of all tumors.^1–5^ These small GTPases function as molecular switches that regulate key signalling pathways controlling cell growth, differentiation, and survival.^6–9^ RAS isoforms are distinguished by the post-translational modifications (PTMs) within their unstructured C-terminal hypervariable regions (HVRs).^10–15^ One isoform, KRAS4B (Kirsten rat sarcoma viral oncogene, hereafter referred to as KRAS), depends primarily on farnesylation for membrane association, whereas another isoform, NRAS (neuroblastoma oncogene), undergoes both farnesylation and palmitoylation.^16^ These lipid anchors drive the recruitment of RAS to biological membranes, where they can assemble into higher-order structures important for downstream signaling activation.^17,18^ Oligomerization of RAS on membrane have been extensively studied using various analytical techniques, showing that RAS forms dimers up to a hexamer,^19–24^ which then enables specific interactions with downstream signaling proteins.^25,26^ An important consequence of RAS oligomerization is the observation that wild-type (WT) RAS isoforms can function as suppressors of tumors driven by oncogenic RAS mutants. For example, gene knock-out mouse model studies showed that the loss of WT KRAS accelerated the downstream signaling and increased the abundance of dimeric KRAS mutants.^27^ Another study using engineered mouse model showed increased the RAS downstream signaling in the absence of NRAS and other RAS isoforms.^28^ Despite conflicting findings of the oligomeric states of RAS on membrane,^29–31^ the disruption of RAS oligomerization is still an attractive therapeutic approach against RAS-driven cancers.^32–34^ This therapeutic relevance highlights the importance of understanding the factors that govern RAS oligomerization on the membrane.

Several factors have been found to affect the RAS oligomerization on membranes, such as lipid composition, nucleotide bound states, and post-translational modifications (PTMs). Multiple studies have shown that RAS possesses selectivity with distinct membrane lipids in isoform specific manners. For example, KRAS oligomers are enriched on phosphatidylserine (PS) containing membranes due to the association of poly-basic domain of KRAS with these lipids.^35–37^ Furthermore, nucleotide bound state is known to be another factor that influences RAS oligomerization. KRAS on membrane is predominantly a monomer in inactive GDP-bound state but forms a dimer when it is in active GTP-bound state.^38,39^ Finally, the differences in the HVRs of RAS isoforms attribute to their distinct behavior on lipid membrane. A recent native mass spectrometry (MS) study revealed that NRAS oligomerization was regulated by PTM, which led to the palmitoylated NRAS favoring the monomeric state meanwhile non-palmitoylated NRAS favors the dimeric state.^40^ In summary, RAS oligomerization on membranes is governed by lipid composition, nucleotide-bound state, and PTMs, which together drive isoform-specific distinct oligomeric behaviors.

Despite these advances to our understanding of RAS oligomerization, prior work has largely been conducted at room temperature, leaving gaps in our knowledge about the behavior of RAS assemblies at physiological temperatures. To better understand biomolecular interactions at physiological temperatures, previous studies have integrated a variable-temperature electrospray ionization apparatus with native MS that regulates solution temperature immediately before ionization, allowing samples to be introduced into the mass spectrometer under defined thermal conditions.^41–51^ This workflow enables direct detection of oligomeric and ligand-bound states that emerge at physiological temperatures but are diminished or absent at room temperature, including a recent study that showed enhanced lipid and metal binding to membrane proteins at physiological temperatures.^52^ Beyond just the observation of differing abundances of oligomeric species with temperature, vt-native MS may also reveal the entropic and enthalpic driving forces behind these interactions to provide greater insight into the fundamental mechanisms governing biomolecular assembly.^43,44^ For example, variations in lipid structure, such acyl chain and headgroup modifications, have been shown to alter thermodynamic strategies for membrane protein-lipid interactions, suggesting solvent rearrangement for interactions involving certain lipid structures.^53^ Using this approach for proteoliposome incorporated RAS proteins, we monitored the oligomerization of KRAS and NRAS on membranes of defined composition across a range of temperatures, revealing temperature-dependent KRAS assembly on select lipid compositions as well as the entropic and enthalpic forces driving RAS oligomerization.

To evaluate if KRAS dimerization is temperature dependent, we reconstituted GTP loaded WT KRAS (hereafter referred to as KRAS) onto 1-palmitoyl-2-oleoyl-*glycero*-3-phosphocholine (POPC) proteoliposomes. These samples were introduced into the mass spectrometer using variable-temperature electrospray ionization (vt-ESI),^41,54^ and RAS proteins were ejected from the liposomes by the application of collisional energy as previously reported.^55^ At room temperature (25 °C), KRAS populated both monomer and dimer species, with the monomer being more abundant (Fig. 1A and Fig. S1A), in agreement with our earlier findings.^40^ Increasing the solution temperature to 30 °C resulted in no change in the monomer and dimer abundances compared to room temperature (Fig. 1B and Fig. S1B). However, at 34 °C, the equilibrium shifted toward predominantly dimeric KRAS (Fig. 1C and Fig. S1C), with further enrichment of the dimeric species at 38 °C (Fig. 1D and Fig. S1D). To assess whether this temperature-dependent assembly of KRAS required a membrane, we introduced DDM (*n*-dodecyl-β-d-maltoside) to solubilize the proteoliposomes. At 25 °C, KRAS in DDM solubilized KRAS was completely monomeric (Fig. 1E and Fig. S2A), consistent with prior observations,^40^ and it remained monomeric even as the temperature increased to 38 °C (Fig. 1F and Fig. S2B). Together, these results show KRAS dimerization requires a membrane, and that this assembly is temperature-dependent on POPC bilayers.

**Fig. 1:**
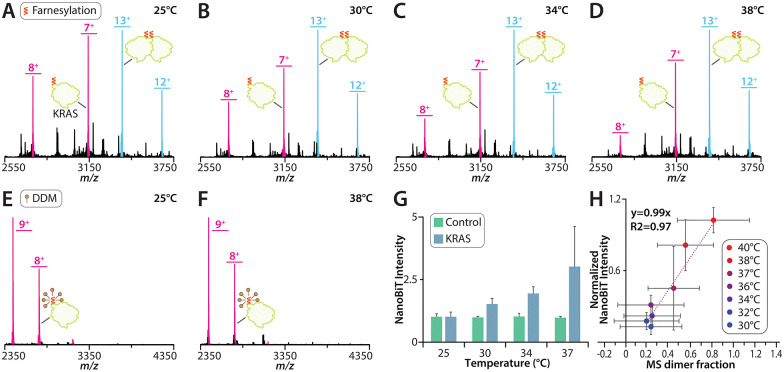
Temperature dependence of KRAS oligomerization on POPC liposomes. (A)–(D) Mass spectra of KRAS incorporated in POPC liposomes collected at temperatures of (A) 25, (B) 30, (C) 34, and (D) 38 °C. Unidentified peaks represent lipid clusters. (E) and (F) Mass spectra of DDM-purified KRAS collected at temperatures of (E) 25 and (F) 38 °C. (G) NanoBiT luminescence signal intensity of KRAS on POPC liposomes collected at temperatures of 25, 30, 34, 37 °C as compared to the control construct that consists of LgBiT fused to SmBiT collected at temperatures of 25, 30, 34, 37 °C. (H) Correlation plot of KRAS dimer mole fraction determined using native MS as compared to the normalized NanoBiT luminescence signal intensity in POPC liposomes.

To corroborate our native MS findings, we employed the solution-based NanoBiT protein-fragment complementation reporter system,^56^ as previously described.^40^ In this assay, a mixture of KRAS genetically fused to either the Large BiT (LgBiT) or the Small BiT (SmBiT) were reconstituted into POPC liposomes. Oligomerization of KRAS brings LgBiT and SmBiT into close proximity, reconstituting an active split nanoluciferase that produces a luminescent signal. Consistent with our native MS results, we observed that increasing temperature led to a corresponding increase in luminescent signal, indicating a higher abundance of KRAS oligomers at elevated temperatures (Fig. 1G). To exclude the possibility that temperature dependence did not originate from the intrinsic interaction between LgBiT and SmBiT alone, we generated a control construct in which LgBiT was genetically fused to SmBiT with a flexible linker. Unlike the constructs containing KRAS, this fusion protein exhibited temperature-independent luminescence (Fig. 1G). The temperature-dependent luminescent signal closely agrees with the fractional abundance of KRAS measured by native MS (Fig. 1H). However, it is important to note that the NanoBiT assay requires genetic fusion of reporter fragments, which may influence KRAS dimerization. Nonetheless, results from both the NanoBiT assay and native MS demonstrate that temperature modulates KRAS oligomerization.

Building on our observation that KRAS dimerization in POPC membranes is temperature dependent, we carried out parallel experiments with KRAS reconstituted into liposomes of varying lipid compositions. In these experiments, we examined POPC membranes doped with 5% 1-palmitoyl-2-oleoyl-*sn-glycero*-3-phosphate (5% POPA liposomes), 10% 1-palmitoyl-2-oleoyl-*sn-glycero*-3-phosphoethanolamine (10% POPE liposomes), or 20% 1-palmitoyl-2-oleoyl-*sn-glycero*-3-phospho-l-serine (20% POPS liposomes), as prior work has shown that these lipid compositions modulate the KRAS monomer–dimer equilibrium at ambient temperature.^40^ KRAS in 5% POPA liposomes did not show any change in equilibrium at higher temperatures (Fig. 2A, B and Fig. S3). Similarly, KRAS in 10% POPE liposomes did not show any change in dimer abundance at elevated temperatures (Fig. 2C, D and Fig. S4). In contrast, the dimer abundance of KRAS on 20% POPS liposomes displayed temperature dependence (Fig. 2E, F and Fig. S5), mirroring the behavior observed for KRAS on POPC liposomes (Fig. 1A–D and Fig. S1). These findings demonstrate temperature- and lipid-dependent dimerization of KRAS (Fig. 2G).

**Fig. 2:**
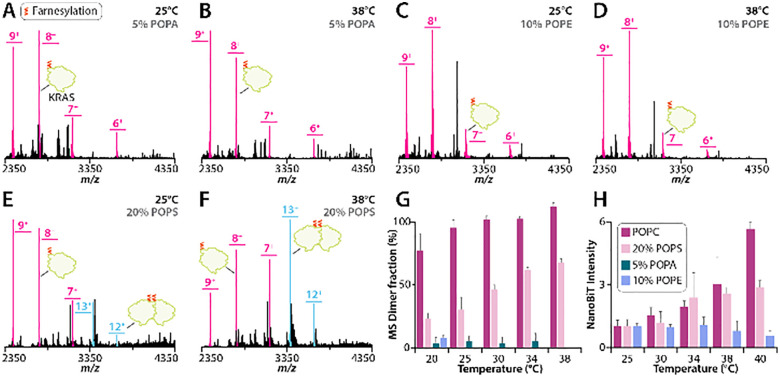
Temperature dependence of KRAS oligomerization in various liposome compositions. (A)–(F) Mass spectra collected of KRAS incorporated in (A) and (B) 5% POPA, (C) and (D) 10% POPE, and (E) and (F) 20% POPS liposome at temperatures of (A), (C) and (E) 25 and (B), (D) and (F) 38 °C. (G) Representative KRAS dimer mole fraction determined using native MS in POPC, 20% POPS, 5% POPA or 10% POPE liposomes collected at temperatures of 20, 25, 30, 34, 38 °C. (H) Representative NanoBiT luminescence signal intensity of KRAS collected at temperatures of 25, 30, 34, 38, 40 °C in POPC, 20% POPS, or 10% POPE liposomes.

To further probe the thermodynamic basis of this temperature-dependent assembly, we performed van’t Hoff analysis of KRAS dimerization on POPC and 20% POPS liposomes. (Fig. S6). Under these experimental conditions, the analysis at 298 K yielded a Δ*H* of 105 ± 5 kJ mol^−1^ and −*T*Δ*S* of −125 ± 5 kJ mol^−1^ for POPC, and Δ*H* of 185 ± 25 kJ mol^−1^ and −*T*Δ*S* of −203 ± 23 J mol^−1^ for 20% POPS liposomes.

These native MS findings were cross validated by the NanoBiT assay. No temperature-dependent change in oligomerization for KRAS on 5% POPA or 10% POPE liposomes was observed. In contrast, temperature-dependence oligomerization of KRAS on POPC or 20% POPS liposomes followed similar trends observed for native MS (Fig. 2H). Together, the NanoBiT and native MS data demonstrate that increasing temperature enhances KRAS oligomerization for only certain lipid compositions.

Next, we focused on GTP-loaded NRAS WT (hereafter referred as NRAS), which is distinct from KRAS by an additional palmitoylation. Consistent with our previous work, purified NRAS contained a mixture of fully (farnesylation and palmitoylation) and partially (farnesylation) modified forms (Fig. 3 and Fig. S7). We compared the monomer and dimer abundance of palmitoylated and non-palmitoylated samples separately. Across all liposome compositions examined (POPC, 10% POPE, and 20% POPS), fully modified NRAS was monomeric whereas the non-palmitoylated species was predominantly dimeric (Fig. 3 and Fig. S8–S10).^40^ The dimeric non-palmitoylated NRAS assembly was not affected by temperature nor dependent on lipid composition (Fig. 3), in contrast to KRAS on POPC (Fig. 1A–D and Fig. S1) and 20% POPS (Fig. 2E, F and Fig. S5) liposomes. These results reveal KRAS and NRAS display different temperature and lipid composition dependencies.

**Fig. 3:**
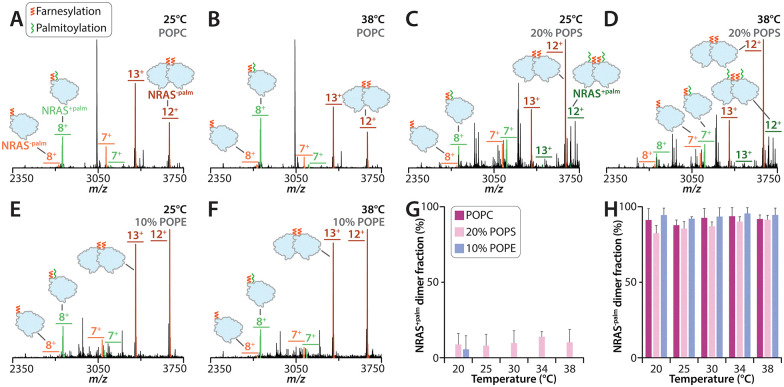
Temperature dependence of NRAS oligomerization in various liposome compositions. (A)–(F) Mass spectra of NRAS incorporated in (A) and (B) POPC, (C) and (D) 20% POPS, and (E) and (F) 10% POPE liposomes at temperatures of (A), (C) and (E) 25 and (B), (D) and (F) 38 °C. Unidentified peaks represent lipid clusters. (G) and (H) NRAS dimer mole fraction determined with native MS in POPC, 20% POPS, or 10% POPE liposomes collected at temperatures of 20, 25, 30, 34, 38 °C with (G) farnesylation and palmitoylation or (H) just farnesylation.

In conclusion, we used vt-ESI native MS and the NanoBiT complementation assay to reveal that KRAS dimerization on membranes is temperature dependent and regulated by lipid composition. Specifically, KRAS dimerization was enhanced at physiological temperatures on POPC and POPS containing liposomes but remained unchanged on POPA and POPE containing liposomes, demonstrating that the temperature dependence of KRAS dimerization is lipid specific. In contrast, NRAS oligomeric states were independent of both temperature and lipid composition, highlighting isoform specific differences in the mechanisms governing RAS assembly on membranes. The entropy driven nature of KRAS dimerization on select lipid compositions implicate solvent, and lipid reorganization are likely key determinants of membrane-dependent KRAS assembly. These findings underscore that prior studies conducted exclusively at room temperature may have underestimated the extent of KRAS dimerization under physiological conditions.

While we focused on well-defined lipid mixtures to isolate the effects of specific headgroups, incorporating additional membrane components, such as cholesterol, warrants further investigation. Approaches that aim to disrupt KRAS oligomerization will need to account for the entropic and lipid-dependent contributions that become more pronounced at physiological temperature. Moreover, the distinct temperature and lipid sensitivities we observe for KRAS compared to NRAS may provide opportunities for isoform-specific targeting. Furthermore, we showed that vt-ESI native MS can be utilized as a versatile platform to directly resolve these temperature-induced equilibrium shifts between lipid environments. Ultimately, these findings provide new insight for understanding how temperature, PTMs, and membrane dynamics influence RAS assemblies.

J. K., S. D. Y., and A. L. designed research; J. K., E. S., S. D. Y., J-Y. C., H. B., J. D., and K. E. performed research; J. K., and A. L. analyzed data; and J. K., and A. L. wrote the paper with input from other authors.

## Conflicts of interest

No potential conflicts of interest were disclosed.

## Supplementary Material

CC-062-D6CC01853J-s001

## Data Availability

The data generated in this study are available within the article and its supplementary information (SI) files. Supplementary information: experimental details, raw and deconvoluted mass spectra on KRAS and NRAS in POPC, 5% POPA, 10% POPE and 20% POPS liposomes at various temperatures, native MS parameters for KRAS and NRAS in DDM and in all liposome compositions, mass table of KRA, NRAS, and related PTMs. See DOI: https://doi.org/10.1039/d6cc01853j.
