# Supplementary material for: Temperature and lipid composition differentially regulate KRAS assemblies on membranes
Source: Chem Commun (Camb). 2026 May 5;62(42):10723–6. doi: 10.1039/d6cc01853j (PMC13170742; doi:10.1039/d6cc01853j)
Supplement: CC-062-D6CC01853J-s001 [file CC-062-D6CC01853J-s001.pdf]

Supplementary Information (SI) for ChemComm.
This journal is © The Royal Society of Chemistry 2026

Supplementary Information

Temperature and Lipid Composition Differentially Regulate KRAS Assemblies on Membranes

Ji Kang,[1] Elena Scott,[1] Sangho D. Yun,[1] Virginia K. James,[1] Jing-Yuan Chang,[1] Hanieh Bahramimoghaddam,[1] James Downing,[1] Kacie Evans,[1] David Russell,[1] Arthur Laganowsky[1*]

1. Department of Chemistry, Texas A&M University, College Station, TX 77843, United States of America

*Corresponding author: alaganowsky@chem.tamu.edu

Table of Contents

**Methods**	S2-S6
**Figure S1.** Deconvoluted mass spectra of KRAS in POPC liposomes.	S7
**Figure S2.** Raw and deconvoluted mass spectra of KRAS with the presence of DDM.	S8
**Figure S3.** Raw and deconvoluted mass spectra of KRAS in 5% POPA liposomes.	S9
**Figure S4.** Raw and deconvoluted mass spectra of KRAS in 10% POPE liposomes.	S10
**Figure S5.** Raw and deconvoluted mass spectra of KRAS in 20% POPS liposomes.	S11
**Figure S6.** van't Hoff plots of KRAS dimerization on (A) POPC and (B) 20% POPS liposomes.	S12
**Figure S7.** Deconvoluted mass spectra of NRAS in POPC liposomes with different PTMs.	S13
**Figure S8.** Deconvoluted mass spectra of NRAS in POPC liposomes.	S14
**Figure S9.** Deconvoluted mass spectra of NRAS in 10% POPE liposomes.	S15
**Figure S10.** Deconvoluted mass spectra of NRAS in 20% POPS liposomes.	S16
**Figure S11.** KRAS in POPC liposomes with various incubation times prior to sample injection to MS.	S17
**Table S1.** MS parameters for KRAS in various liposome compositions.	S18
**Table S2.** MS parameters for NRAS in various liposome compositions.	S19
**Table S3.** MS parameters for RAS proteins without liposome.	S20
**Table S4.** Expected masses of PTMs on RAS proteins.	S21
**Table S5.** Theoretical and observed masses of RAS proteins.	S22
**References**	S23

Methods

Expression and purification of full-length RAS proteins. KRAS and NRAS proteins were expressed and purified using previously described protocol.[1] The KRAS4B (hereafter referred as to KRAS) gene (residues 1–188, UniProt P01116-2) was obtained from the Frederick National Laboratory for Cancer Research (MD, USA) as part of the construct His6-MBP-TEV-G-Hs.KRAS (1–188). This gene was similarly subcloned into the pACEBac1 vector with an N-terminal His6-tag and a TEV-cleavable mCherry fusion. The NRAS gene (residues 1–189, UniProt P01111) were subcloned from the RAS mutant clone collection (Addgene, Cat. 1000000089) into the pACEBac1 vector (Geneva Biotech), incorporating an N-terminal His6-tag followed by a TEV protease-cleavable mCherry. Each plasmid was transformed into DE100 cells (Frederick National Laboratory for Cancer Research) to generate recombinant bacmids, which were used to transfect Spodoptera frugiperda (Sf9) cells using PEI-Max (Polysciences) to produce P1 virus.[2] Proteins were expressed in Trichoplusia ni (Tni) cells for 72 hours at 27 °C with shaking after baculovirus infection. Cells were harvested by centrifugation at 4,000 ×g for 10 minutes and stored at −80 °C until further use. Frozen pellets were resuspended in the lysis buffer (300 mM NaCl, 20mM HEPES, 5mM MgCl2, pH 7.4) and lysed using a Microfluidics M-110P microfluidizer at 25,000 psi. Cell debris were removed by centrifugation (20,000 ×g, 25 minutes, 4 °C), Membrane were isolated by ultracentrifugation (100,000 ×g, 2 hours, 4 °C) and resuspended in the same buffer as lysis buffer, with optional storage at -80 °C for the future use. To extract the protein, membranes were incubated with 1% (w/v) n-dodecyl-β-D-maltoside (DDM) for 2 hours at 4 °C. The extract was clarified by centrifugation (20,000 ×g, 10 minutes, 4 °C) and filtered (0.45 µm syringe filter, Pall Corp.). Filtered extract was loaded onto a 2 mL Ni-NTA Superflow resin (Qiagen) gravity-flow column pre-equilibrated with NHA-DDM buffer (300 mM NaCl, 20 mM HEPES, 20 mM imidazole, 5 mM $MgCl_2$, 0.025% DDM, pH 7.4). After washing, the proteins were eluted with NHB-DDM buffer (NHA-DDM with containing 500 mM imidazole). Eluted proteins were desalted using a 30 kDa MWCO concentrator (MilliporeSigma) with NHA-DDM. The His6-tag was removed by overnight TEV protease digestion at 4 °C. The cleaved protein was passed again through Ni-NTA resin (Qiagen) gravity-flow column to separate the cleaved tag and tag-less protein. The

flowthrough was collected and concentrated using a 10 kDa MWCO (Molecular Weight Cut-off) concentrator (MilliporeSigma).

For the Large BiT (LgBiT)-KRAS construct, the KRAS gene (residues 1–188, UniProt P01116-2) was cloned into pACEBac1 (Geneva Biotech) with the following architecture: sequence His6–SSG–HRV3C–GG–LgBiT–GSGGGSGT–TEV–S–KRAS. The Small BiT (SmBiT)-KRAS construct was generated similarly with the sequence of His6–SSG–HRV3C–GG–VTGYRLFEEIL–GSGGGSGT–TEV–S–KRAS, where VTGYRLFEEIL corresponds to the SmBiT peptide sequence. Expression and purification of both constructs followed the same procedure described for full-length RAS proteins, except the His6-tag was not cleaved.

For use as a control in the NanoBiT assay, the LgBiT and SmBiT peptide sequences were cloned into a pET28 vector to generate a control construct in which LgBiT was genetically fused to SmBiT in the presence of a flexible linker with the sequence of Strep II-LgBiT-TEV-GG-SmBiT. The plasmid was expressed in *E. coli* Rosetta 2 (DE3) cells cultured in LB medium supplemented with 50 µg/mL kanamycin. Protein expression was induced with 0.5 mM IPTG when cultures reached an OD_{600} of 0.6–0.8, followed by overnight incubation at 18 °C. Cells were harvested by centrifugation at 5,000 × g, and resulting pellets were stored at −80 °C until further use. Thawed cells were resuspended in buffer A (50 mM Tris, 150 mM NaCl, pH 7.4) and lysed and clarified as described above. The clarified lysate was loaded onto a Strep Tag affinity column (4 mL bed volume, prepared in-house with Strep-Tactin Sepharose resin, iba) equilibrated in equilibrated with buffer A. Bound protein was eluted with buffer B (buffer A with 3 mM desthiobiotin) and concentrated using a 10 kDa MWCO centrifugal filter (Millipore). All protein solutions were supplemented with 20% (w/v) glycerol, flash-frozen, and stored at −80 °C.

GTP loading of RAS proteins. Purified KRAS, NRAS, LgBiT-KRAS, and SmBiT-KRAS were all loaded with GTP following the established protocol.[3] Proteins were incubated with 30- to 50-fold molar excess of GTP in the presence of 10 mM EDTA for 3 hours at 4 °C. The reaction mixture was then supplemented with 10 mM $MgCl_2$ and incubated for 30 minutes at 4 °C. Excess nucleotide, $MgCl_2$, and EDTA were removed using a Micro Bio-Spin 6 desalting column (Bio-Rad). All proteins were GTP-loaded prior to preparation of proteoliposome and NanoBiT assay.

Preparation of RAS proteins containing proteolipsomes. Proteoliposomes containing KRAS, NRAS, LgBiT-KRAS and SmBiT-KRAS were prepared following a previously reported protocol with minor modifications.[4] Lipid stocks (Avanti Polar Lipids) in chloroform were dried under the stream of nitrogen and washed with pentane (Sigma Aldrich) by adding ~2 mL of pentane into the HPLC sample vial (Agilent). Dried lipid stocks were placed in a vacuum chamber overnight to form thin lipid films. Lipid films were rehydrated in 1mL of water to a final concentration of 20 mM. For POPC liposomes, rehydrated POPC lipid stocks were diluted with RAS proteins lysis buffer (300 mM NaCl, 20mM HEPES, 5mM MgCl2, pH 7.4) to reach final lipid concentration of 10 mM. For liposomes containing mixed lipids, lipid stocks were mixed to achieve the desired molar ratios prior to the initial drying process using nitrogen stream. For example, POPC liposome containing 10% POPE would have 2 mM POPE and 18 mM POPC before being diluted to 10 mM final lipid concentration. The mixture of rehydrated lipid with the lysis buffer was extruded through a 50 nm polycarbonate membrane (Cytiva) with multiple passes, or until the solution appeared translucent, to produce large unilamellar vesicles (LUV). To solubilize the LUVs, DDM was added at twice the critical micelle concentration (CMC) to the extruded lipid, and the lipid-detergent mixture was rotated at 4 °C for 2 hours or more. 10 µM of GTP-loaded KRAS or NRAS protein was then added to the lipid-detergent mixture to reach final protein-to-lipid molar ratio of 1:500. For the proteoliposome containing NanoBiT fused RAS proteins, the mixture of final concentration of 5uM LgBiT-KRAS and 5uM SmBiT-KRAS was added. The resulting protein-lipid mixture was diluted with the lysis buffer to a final lipid concentration of 5 mM and incubated for 1 hour at 4 °C under rotation to allow incorporation of the protein into the lipid phase. After the incubation for proteins to be incorporated into liposomes, detergent removal was achieved by adding washed Bio-Beads (Bio-Rad) and rotating the mixture for 1 hour at 4 °C. The resulting proteoliposomes were carefully collected to avoid Bio-Beads contamination and subsequently extruded through a 100 nm membrane (Cytiva) to generate uniform unilamellar vesicles. To remove residual detergent and salts, the proteoliposomes were dialyzed overnight at 4 °C against 1 L of 200 mM ammonium acetate (pH 7.4) using a 10 kDa MWCO dialysis membrane (Thermo Scientific).

NanoBiT assay. SmBiT and LgBiT fused KRAS were incorporated into solubilized lipids at a protein-to-lipid molar ratio of GTP-SmBiT-KRAS: GTP-LgBiT-KRAS: lipid 1:1:2000, with a combined

GTP-SmBiT KRAS and GTP-LgBiT KRAS concentration of 10 μM. Formation of GTP-SmBiT KRAS and GTP-LgBiT KRAS incorporated liposome was achieved by overnight dialysis at 4 °C against 1 L of 200 mM ammonium acetate (pH 7.4) using a 10 kDa MWCO dialysis membrane (Thermo Scientific). For luminescence measurements, proteoliposomes were diluted to a final protein concentration of 0.25 μM in the presence of 5 μM coelenterazine 400a (Nanolight Technologies) and incubated for 1 minute at desired temperature before recording. The temperatures of 25, 30, 32, 34, 36, 37, 38, and 40 °C were used for GTP loaded KRAS in POPC, 20% POPS, and 5% POPA liposomes, and temperatures of 25, 30, 34, and 37 °C were used for the control. Luminescence emission between 470 ± 20 nm was monitored.

Native mass spectrometry (MS). All Native MS data were collected on an Exactive Plus with an Extended Mass Range Orbitrap mass spectrometer (Thermo Scientific). Proteoliposome samples containing total protein concentration of 10 μM were dialyzed against 200 mM ammonium acetate and were analyzed. Native MS data acquisition was operated under the specified tuning parameters (**Table S1-S2**). For experiments without liposomes, detergent solubilized proteins were buffer exchanged into 200 mM ammonium acetate with 0.025% DDM in a centrifugal desalting column (Micro Bio-Spin 6, Bio-Rad) and analyzed using native MS under the specified tuning parameters in **Table S3**. Expected post-translational modifications (PTMs) for KRAS and NRAS are provided in **Table S4**. KRAS proteins with and without liposome are fully modified and loaded with GTP. NRAS proteins with and without liposome are either fully modified or in the absence of palmitoylation. Theoretical and measured masses for KRAS and NRAS proteins shown in this manuscript are provided in **Table S5**. Variable temperature nano electrospray ionization experiments were performed as previously described.[5] To confirm that RAS oligomer equilibrium was reached, we monitored the monomer-to-dimer ratio during incubation at a fixed temperature for 1–10 min and found no significant differences between 1 and 10 min incubation times across all temperatures examined (**Figure S10**). All data were collected in triplicate.

Native MS data analysis. Native MS data were deconvoluted using UniDec.[6] The signal intensities of monomeric RAS (m) and dimeric RAS (d) were converted into monomer molar fraction and dimer molar fraction for a given temperature, using equations 1 and 2, respectively.

$$Monomer\ molecular\ fraction\ (m) = \frac{M}{M + D}$$ *Equation 1*

$$Dimer\ molecular\ fraction\ (d) = \frac{D}{M + D}$$ *Equation 1*

The interaction between RAS monomers and dimers is dependent on the equilibrium association constant (K_A). K_A value was calculated with m and d calculated from equations 1 and 2, respectively, using the van't Hoff equation described in equation 3.

$$K_A = \frac{d \times total\ protein\ concentration}{(m \times total\ protein\ concentration)^2}$$ *Equation 3*

Van't Hoff analysis[7] was used to determine thermodynamic parameters, enthalpy (ΔH) and entropy (Δ*S)*, based on equation:

$$lnK_A = -\frac{\Delta H}{RT} + \frac{\Delta S}{R}$$ *Equation 4*

Supplementary Figures

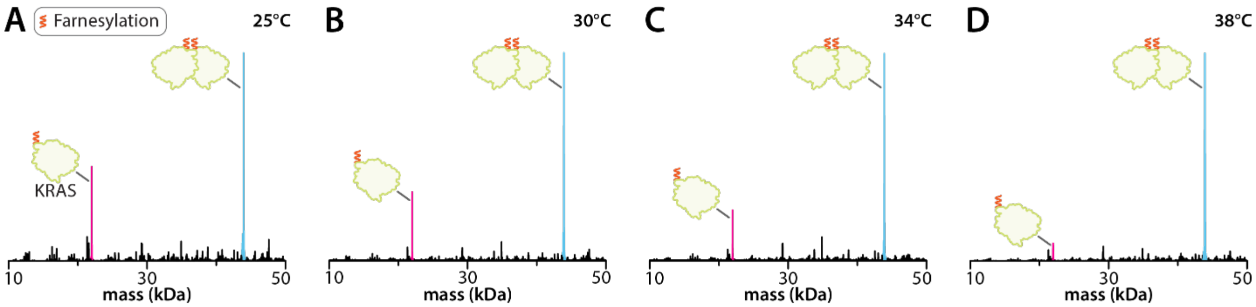

Figure S1. A-D) Deconvoluted mass spectra of KRAS in POPC liposomes at temperatures ranging from 25 to 40 °C.

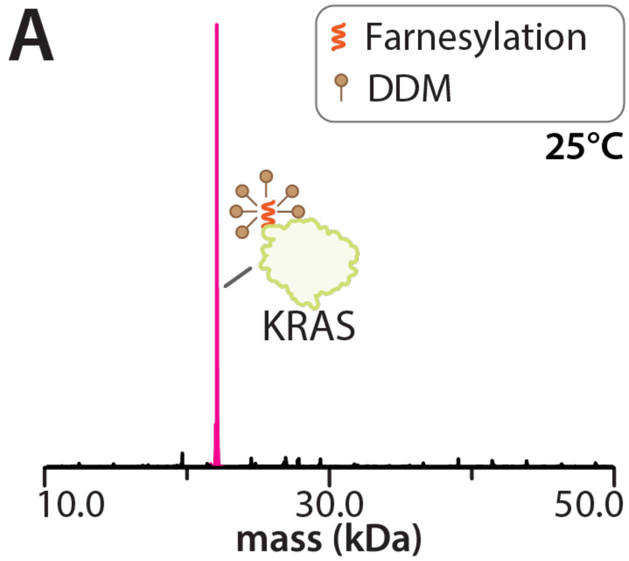

Figure S2. Deconvoluted mass spectra of KRAS with the presence of DDM at temperatures of (A) 25 °C and (B) 38 °C.

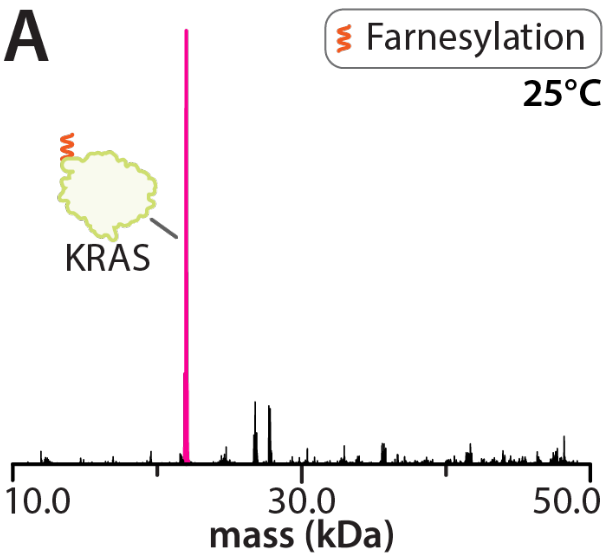

Figure S3. Deconvoluted mass spectra of KRAS in 5% POPA liposome at temperatures of (A) 25 °C and (B) 38 °C.

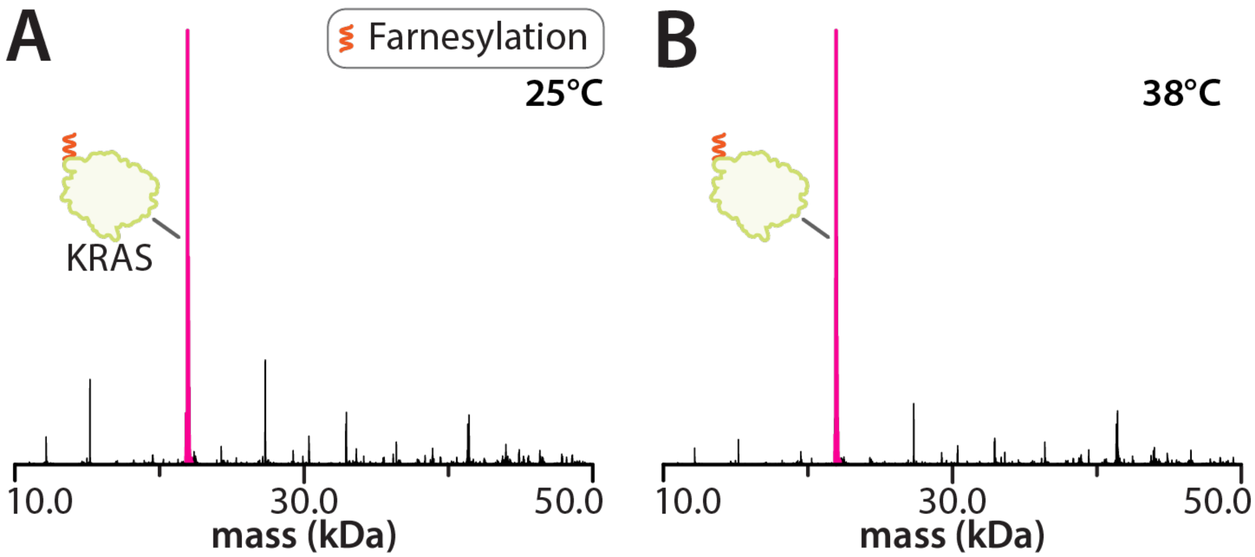

Figure S4. Deconvoluted mass spectra of KRAS in 10% POPE liposome at temperatures of (A) 25 °C and (B) 38 °C.

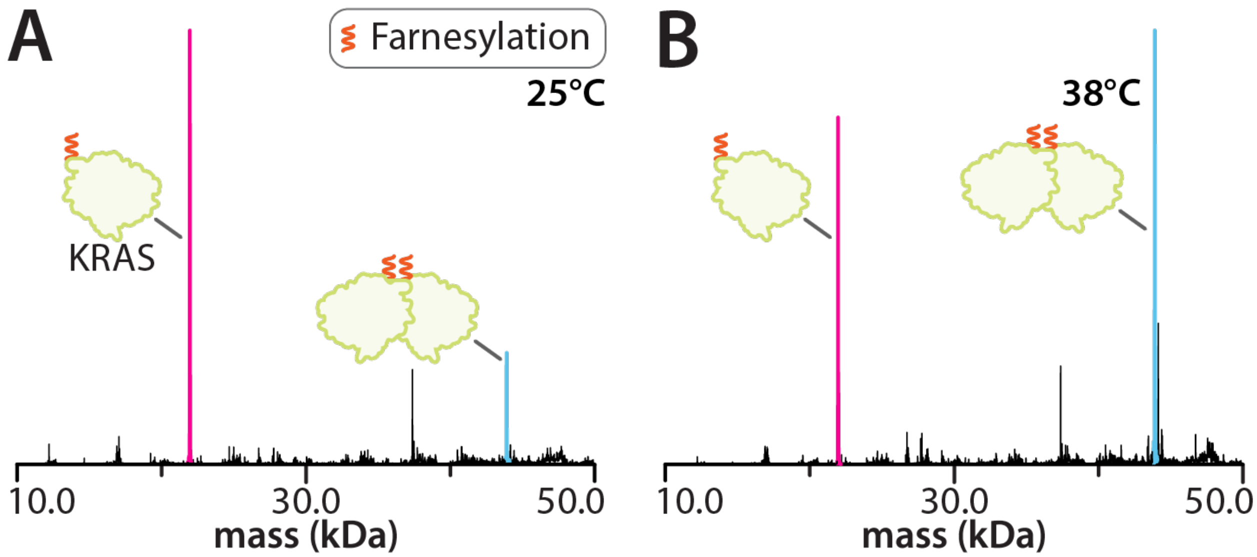

Figure S5. Deconvoluted mass spectra of KRAS in 20% POPS liposome at temperatures of (A) 25 °C and (B) 38 °C.

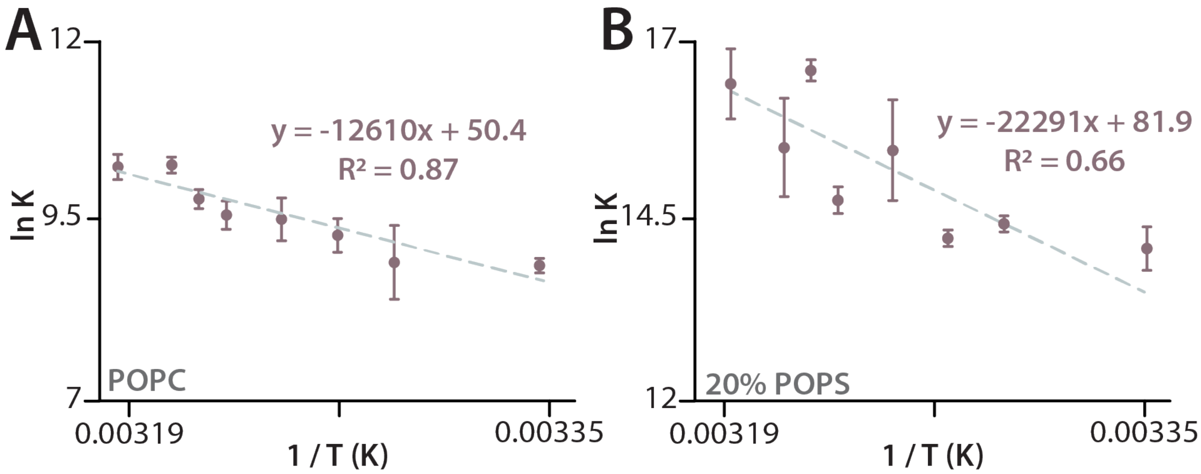

Figure S6. van't Hoff plots of KRAS dimerization on (A) POPC and (B) 20% POPS liposomes.

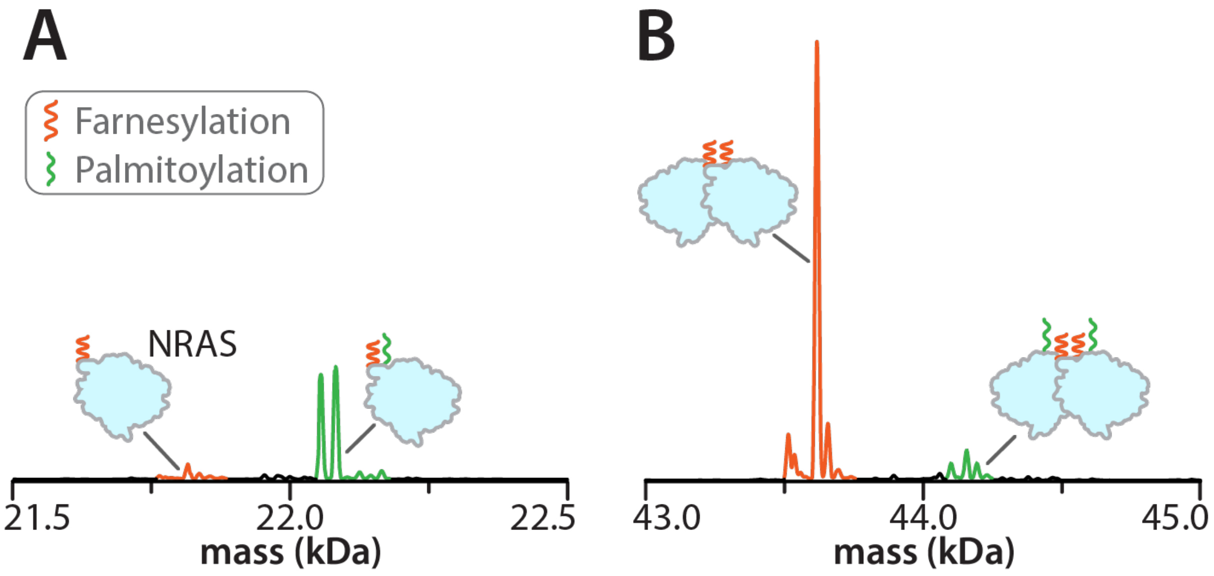

Figure S7. Deconvoluted mass spectra of (A) monomer and (B) dimer farnesylated and palmitoylated NRAS in POPC liposomes collected at 25° C.

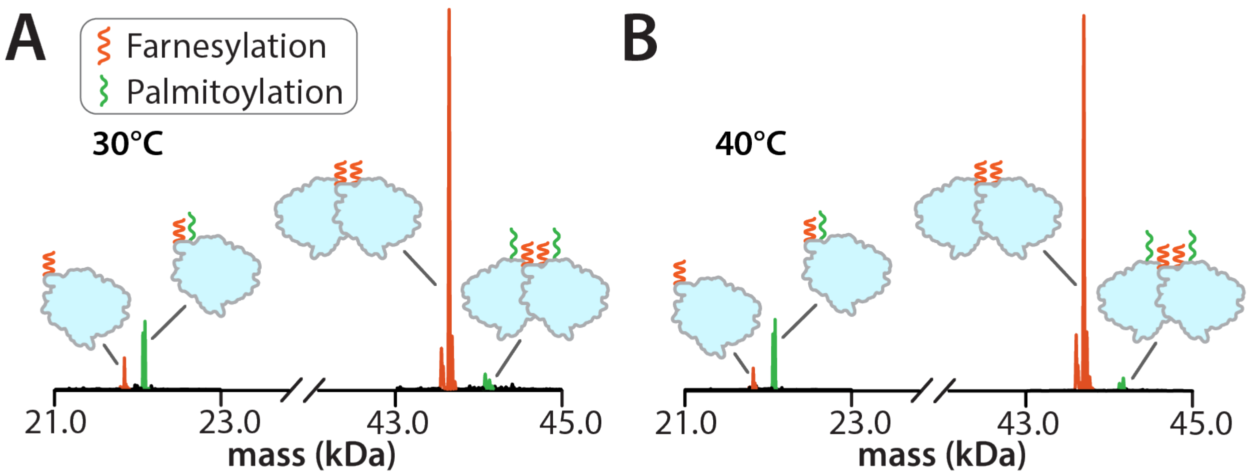

Figure S8. Deconvoluted mass spectra of NRAS in POPC liposomes at temperatures of (A) 25 °C and (B) 38 °C.

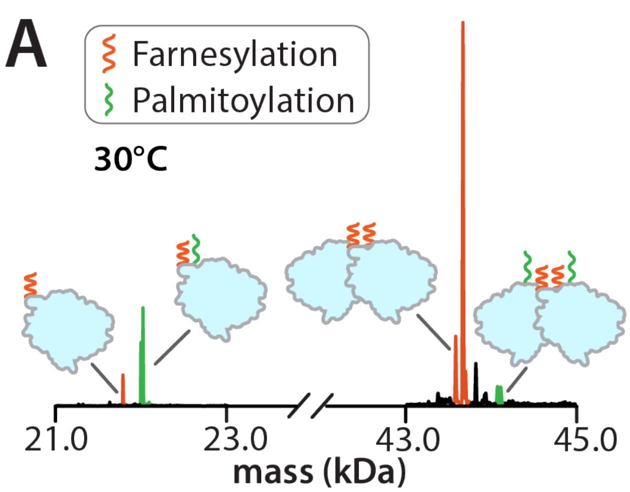

Figure S9. Deconvoluted mass spectra of NRAS in 10% POPE liposomes collected at temperatures of (A) 25 °C and (B) 38 °C.

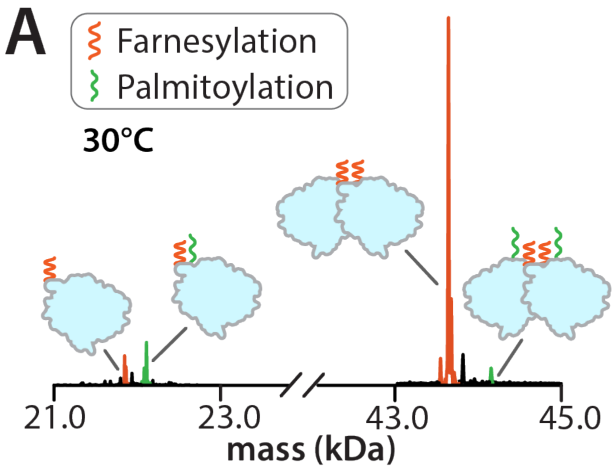

Figure S10. Deconvoluted mass spectra of NRAS in 20% POPS liposomes at temperatures of (A) 25 °C and (B) 38 °C.

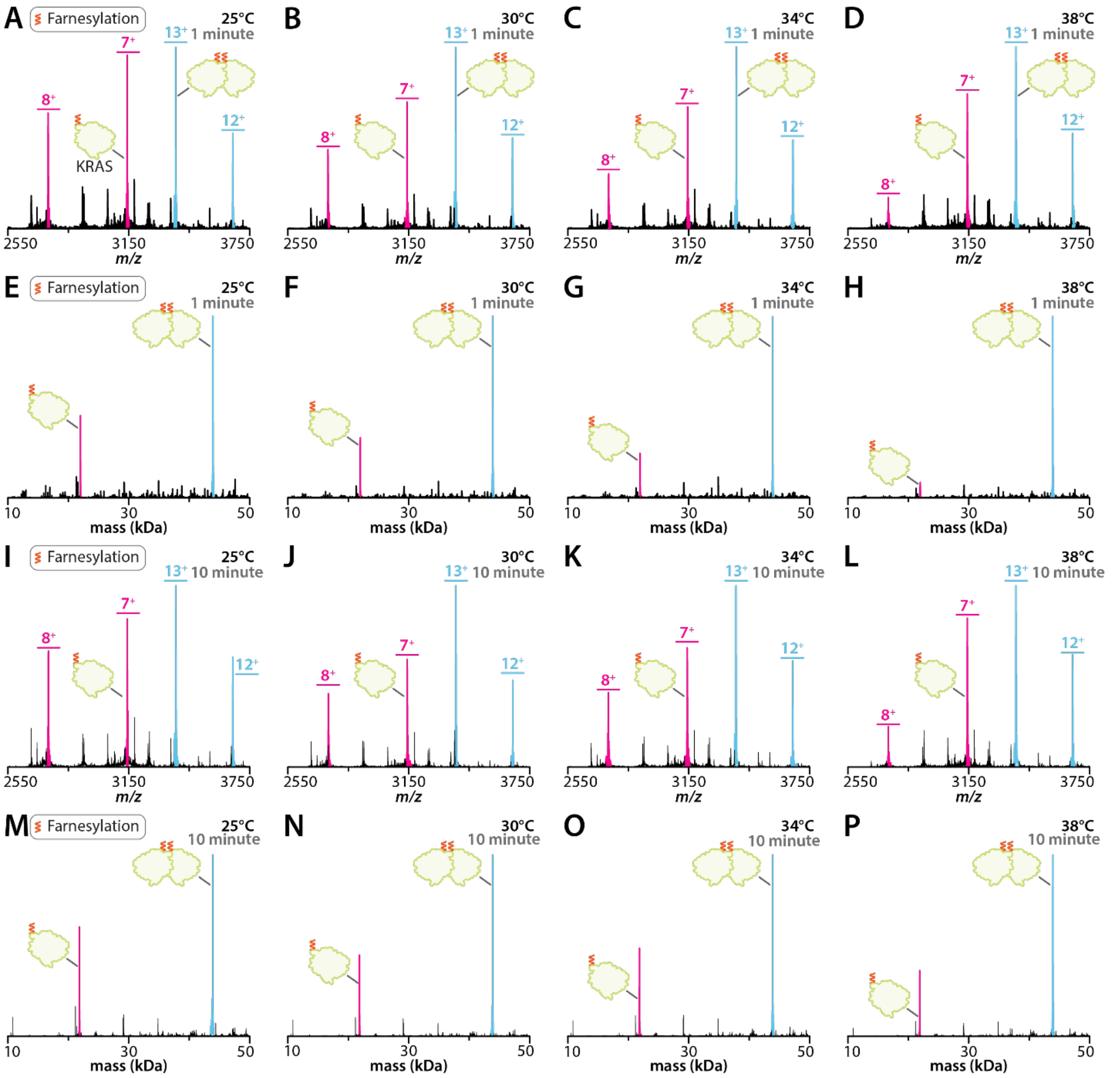

Figure S11. A-D) Raw mass spectra of KRAS in POPC liposome at temperatures ranging from 25 to 40 °C, collected after one minute (A-D) and ten-minute (I-L) incubation at each temperature. Deconvoluted mass spectra for A-D and I-L are provided in E-H and M-P, respectively.

Table S1. MS parameters for KRAS in various liposome compositions.

Parameters	***POPC***	***5% POPA***	***10% POPE***	***20% POPS***
m/z range	2310 - 7000	2310-7000	2310-7000	2310 - 7000
Resolution	17500	17500	17500	17500
In-source CID (V)	70	65	65	30
HCD energy (eV)	40	25	25	40
Capillary temperature (°C)	200	200	200	200
Spray voltage (kV)	1.1-1.5	1.1-1.5	1.1-1.5	1.1-1.5
Source DC offset (V)	25	25	25	25
Inject flatapole DC (V)	15.5	15.5	15.5	15.5
Inter flatapole lens (V)	7	7	7	9
Bent flatapole DC (V)	6	8.5	8.5	3
Transfer multipole DC (V)	5	2.9	2.9	5
C-Trap entrance lens tune offset (V)	6	6.5	6.5	4
UHV pressure (mbar)	4.01×10^{-10}	3.98×10^{-10}	3.98×10^{-10}	4.50×10^{-10}

Table S2. MS parameters for NRAS in various liposome compositions.

Parameters	***POPC***	***10% POPE***	***20% POPS***
m/z range	2310-3750	2310-7000	2310-7000
Resolution	17,500	17,500	17,500
In-source CID (V)	60	95	80
In-Source CE (eV)	70	70	100
Source temperature (°C)	200	200	200
Capillary voltage (kV)	1.5	1.6	1.5
Source DC offset (V)	20	25	25
Inject flatapole DC (V)	15.5	16.5	16.5
Inter flatapole lens (V)	8	9	9.5
Bent flatapole DC (V)	14.7	10	6.5
Transfer multipole DC (V)	8	5	5
C-Trap entrance lens tune offset (V)	2	4	4
UHV pressure (mbar)	4.19×10^{-10}	4.09×10^{-10}	5.56×10^{-10}

Table S3. MS parameters for KRAS and NRAS in detergent.

Parameters	***Value***
m/z range	1600-6000
Resolution	17,500
In-source CID (V)	30 - 40
In-Source CE (eV)	40 - 60
Source temperature (°C)	200
Capillary voltage (kV)	1.1 - 1.5
Source DC offset (V)	25 - 35
Inject flatapole DC (V)	15 - 18
Inter flatapole lens (V)	9 - 12
Bent flatapole DC (V)	0 - 5
Transfer multipole DC (V)	0 - 4
C-Trap entrance lens tune offset (V)	0 - 2
UHV pressure (mbar)	4 -5 × 10^{-10}

Table S4. Expected post-translational modifications (PTMs) and mass differences (Δ Dalton) as compared to the unmodified protein.

Protein	***PTM***	***Δ Dalton***
KRAS-GTP	S-Farnesylation (Farn)	204
	Methylation (met)	14
	CAAX Cleavage	-343
NRAS-GTP	S-Farnesylation (Farn)	204
	Palmitoylation (Palm)	238
	Methylation (met)	14
	CAAX Cleavage	-343

Table S5. Theoretical and observed masses for the RAS proteins.

Protein	Theoretical Mass (Da)	Observed Mass (Da)	Δ Dalton
*KRAS ● Farn ● met (GTP ●Mg^{2+}) **	21987	21989	1.8
*NRAS ● Farn ● met (GTP ●Mg^{2+}) **	21820	21819	0.79
*NRAS ● Farn ● Palm ● met (GTP ●Mg^{2+}) **	22058	22057	0.79

*Theoretical masses have the CAAX cleavages applied.

References

1 Yun, S. D. *et al.* Capturing RAS oligomerization on a membrane. *Proc Natl Acad Sci U S A* **121**, e2405986121 (2024). https://doi.org/10.1073/pnas.2405986121

2 Scholz, J. & Suppmann, S. A new single-step protocol for rapid baculovirus-driven protein production in insect cells. *BMC Biotechnol* **17**, 83 (2017). https://doi.org/10.1186/s12896-017-0400-3

3 Moghadamchargari, Z. *et al.* Intrinsic GTPase Activity of K-RAS Monitored by Native Mass Spectrometry. *Biochemistry* **58**, 3396-3405 (2019). https://doi.org/10.1021/acs.biochem.9b00532

4 Zhu, Y. *et al.* Native mass spectrometry of proteoliposomes containing integral and peripheral membrane proteins. *Chem Sci* **14**, 14243-14255 (2023). https://doi.org/10.1039/d3sc04938h

5 McCabe, J. W. *et al.* Variable-Temperature Electrospray Ionization for Temperature-Dependent Folding/Refolding Reactions of Proteins and Ligand Binding. *Anal Chem* **93**, 6924-6931 (2021). https://doi.org/10.1021/acs.analchem.1c00870

6 Marty, M. T. *et al.* Bayesian deconvolution of mass and ion mobility spectra: from binary interactions to polydisperse ensembles. *Anal Chem* **87**, 4370-4376 (2015). https://doi.org/10.1021/acs.analchem.5b00140

7 van't Hoff, M. J. H. Etudes de dynamique chimique. *Recueil des Travaux Chimiques des Pays-Bas* **3**, 333-336 (1884). https://doi.org/10.1002/recl.18840031003
